# Rejection odds and rejection ratios: A proposal for statistical practice in testing hypotheses^⋆^

**DOI:** 10.1016/j.jmp.2015.12.007

**Published:** 2016-02-05

**Authors:** M.J. Bayarri, Daniel J. Benjamin, James O. Berger, Thomas M. Sellke

**Affiliations:** a Universitat de València, Spain; b University of Southern California, United States; c Duke University, United States; d Purdue University, United States

**Keywords:** Odds, Bayesian, Frequentist, Bayes factors

## Abstract

Much of science is (rightly or wrongly) driven by hypothesis testing. Even in situations where the hypothesis testing paradigm is correct, the common practice of basing inferences solely on *p*-values has been under intense criticism for over 50 years. We propose, as an alternative, the use of the odds of a correct rejection of the null hypothesis to incorrect rejection. Both pre-experimental versions (involving the power and Type I error) and post-experimental versions (depending on the actual data) are considered. Implementations are provided that range from depending only on the *p*-value to consideration of full Bayesian analysis. A surprise is that all implementations – even the full Bayesian analysis – have complete frequentist justification. Versions of our proposal can be implemented that require only minor modifications to existing practices yet overcome some of their most severe shortcomings.

## 1. Introduction

In recent years, many sciences – including experimental psychology – have been embarrassed by a growing number of reports that many findings do not replicate. While a variety of factors contribute to this state of affairs, a major part of the problem is that conventional statistical methods, when applied to standard research designs in psychology and many other sciences, are too likely to reject the null hypothesis and therefore generate an unintentionally high rate of false positives. A number of alternative statistical methods have been proposed, including several in this special issue, and we are sympathetic to many of these proposals. In particular we are highly sympathetic to efforts to wean the scientific community away from an over-reliance on hypothesis testing, with utilization of often-more-relevant estimation and prediction techniques.

Our goal in this paper is more modest in scope: we propose a range of modifications – several *relatively minor* – to existing statistical practice in hypothesis testing that we believe would immediately fix some of the most severe shortcomings of current methodology. The minor modifications would not require any changes in the statistical tests that are commonly used, and would rely only on the most basic statistical concepts and tools, such as significance thresholds, *p*-values, and statistical power. With *p*-values and power calculations in hand (obtained from standard software in the usual way), the additional calculations we recommend can be carried out with a calculator.

In developing and justifying these simple modifications of standard methods, we also discuss additional tools that are available from Bayesian statistics. While these can provide considerable additional benefit in a number of settings, significant improvements in the testing paradigm can be made even without them.

We study the standard setting of precise hypothesis testing.^1^ We can observe data ***x*** from the density *f* (***x*** | *θ*). We consider testing

(1) $$H_{0}:\theta =\theta_{0}\;\;\;\text{versus}\;\;\;H_{1}:\theta \neq \theta_{0}.$$

Our proposed approach to hypothesis testing is based on consideration of the odds of correct rejection of *H*_0_ to incorrect rejection. This ‘rejection odds’ approach has a dual frequentist/Bayesian interpretation, and it addresses four acknowledged problems with common practices of statistical testing:

1. Failure to incorporate considerations of power into the interpretation of the evidence.
2. Failure to incorporate considerations of prior probability into the design of the experiment.
3. Temptation to misinterpret *p*-values in ways that lead to overstating the evidence against the null hypothesis and in favor of the alternative hypothesis.
4. Having optional stopping present in the design or running of the experiment, but ignoring the stopping rule in the analysis.

There are a host of other problems involving testing, such as the fact that the size of an effect is often much more important than whether an effect exists, but here we only focus on the testing problem itself. Our proposal – developed throughout the paper and summarized in the conclusion – is that researchers should report what we call the ‘pre-experimental rejection ratio’ when presenting their experimental design, and researchers should report what we call the ‘post-experimental rejection ratio’ (or Bayes factor) when presenting their experimental results.

In Section 2, we take a pre-experimental perspective: for a given anticipated effect size and sample size, we discuss the evidentiary impact of statistical significance, and we consider the problem of choosing the significance threshold (the region of results that will lead us to reject *H*_0_). The (pre-experimental) ‘rejection ratio’ *R_pre_*, the ratio of statistical power to significance threshold (i.e., the ratio of the probability of rejecting under *H*_1_ and *H*_0_, respectively), is shown to capture the strength of evidence in the experiment for *H*_1_ over *H*_0_; its use addresses Problem #1 above.

How much a researcher should believe in *H*_1_ over *H*_0_ depends not only on the rejection ratio but also on the prior odds, the relative prior probability of *H*_1_ to *H*_0_. The ‘pre-experimental rejection odds,’ which is the overall odds in favor of *H*_1_ implied by rejecting *H*_0_, is the product of the rejection ratio and the prior odds. When the prior odds in favor of *H*_1_ are low, the rejection ratio need to be greater in order for the experiment to be equally convincing. This line of reasoning, which addresses Problem #2, implies that researchers should adopt more stringent significance thresholds (and generally use larger sample sizes) when demonstrating surprising, counterintuitive effects. The logic underlying the pre-experimental odds suggests that the standard approach in many sciences (including experimental psychology) – accepting *H*_1_ whenever *H*_0_ is rejected at a conventional 0.05 significance threshold – can lead to especially misleading conclusions when power is low or the prior odds is low.

In Section 3, we turn to a post-experimental perspective: once the experimental analysis is completed, how strong is the evidence implied by the observed data? The analog of the pre-experimental odds is the ‘post-experimental odds’: the prior odds times the Bayes factor. The Bayes factor is the ratio of the likelihood of the observed data under *H*_1_ to its likelihood under *H*_0_; for consistency in notation (and because of a surprising frequentist interpretation that is observed for this ratio), we will often refer to the Bayes factor as the ‘post-experimental rejection ratio,’ *R_post_*.

Common misinterpretations of the observed *p*-value (Problem #3) are that it somehow reflects the error probability in rejecting *H*_0_ (see Berger, 2003; Berger, Brown, & Wolpert, 1994) or the related notion that it reflects the likelihood of the observed data under *H*_0_. Both are very wrong. For example, it is sometimes incorrectly said that *p* = 0.05 means that there was only a 5% chance of observing the data under *H*_0_. (The correct statement is that *p* = 0.05 means that there was only a 5% chance of observing a test statistic as extreme or more extreme as its observed value under *H*_0_ – but this correct statement is not very useful because we want to know how strong the evidence is, given that we actually observed the value of the test statistic that we did.) Given this misinterpretation, many researchers dramatically overestimate the strength of the experimental evidence for *H*_1_ provided by a *p-*value. The Bayes factor has a straightforward interpretation as the strength of the evidence in favor of *H*_1_ relative to *H*_0_, and thus its use can avoid the misinterpretations that arise from reliance on the *p*-value.

The Bayes factor approach has been resisted by many scientists because of two perceived obstacles. First, determination of Bayes factors can be difficult. Second, many are uneasy about the subjective components of Bayesian inference, and view the familiar frequentist justification of inference to be much more comforting. The first issue is addressed in Section 3.2, where we discuss the ‘Bayes factor bound’ 1/[−*ep* log *p*] (from Sellke, Bayarri, & Berger, 2001 and Vovk, 1993). This bound is the *largest* Bayes factor in favor of *H*_1_ that is possible (under reasonable assumptions). The Bayes factor bound can thus be interpreted as a best-case scenario for the strength of the evidence in favor of *H*_1_ that can arise from a given *p*-value. Even though it favors *H*_1_ amongst all (reasonable) Bayesian procedures, it leads to far more conservative conclusions than the usual misinterpretation of *p*-values; for example, a *p*-value of 0.05 only represents *at most* 2.5 : 1 evidence in favor of *H*_1_. The ‘post-experimental odds bound’ can then be calculated as the Bayes factor bound times the prior odds.

In Section 3.3, we address the frequentist concerns about the Bayes factor. In fact, we show that in our setting, using the Bayes factor is actually a fully frequentist procedure – and, indeed, we argue that it is actually a much better frequentist procedure than that based on the *p*-value or on the pre-experimental rejection ratio. Our result that the Bayes factor has a frequentist justification is novel to this paper, and it is surprising because the Bayes factor depends on the prior distribution for the effect size under *H*_1_. We point out the resolution to this apparent puzzle: the prior distribution’s role is to prioritize where to maximize power, while the procedure always maintains frequentist error control for the rejection ratio that is analogous to Type I frequentist error control.

Our result providing a frequentist justification for the Bayes factor helps to unify the pre-experimental odds with the post-experimental odds. It also provides a bridge between frequentist and Bayesian approaches to hypothesis testing.

In Section 4, we address the practical question of how to choose the priors that enter into the calculation of the Bayes factor and post-experimental odds. We enumerate a range of options. Researchers may prefer one or another of these options, depending on whether or not they are comfortable with statistical modeling, and whether or not they are willing to adopt priors that are subjective. Since the options cover many situations, we argue that there is really no practical barrier to reporting the Bayes factor, or at least the Bayes factor bound. For example, reporting the Bayes factor bound does not require specifying any prior, and it is simple to calculate from just the *p*-value associated with standard hypothesis tests. While the Bayes factor bound is not ideal as a summary of the evidence since it is biased against the null, we believe its reporting would still lead to more accurate interpretations of the evidence than reporting the *p*-value alone.

In Section 5, we show that the post-experimental odds approach has the additional advantage that it overcomes the problems that afflict *p*-values caused by optional stopping in data collection. A common practice is to collect some data, analyze it, and then collect more data if the results are not yet statistically significant (John, Loewenstein, & Prelec, 2012). There is nothing inherently wrong with such an optional stopping strategy; indeed, it is a sensible procedure when there are competing demands on a limited subject pool. However, in the presence of optional stopping, it is well-known that *p*-values calculated in the usual way can be extremely biased (e.g., Anscombe, 1954). Under the null hypothesis, there is a greater than 5% chance of a statistically significant result when there is more than one opportunity to get lucky. (Indeed, if scientists were given unlimited research money and allowed to ignore optional stopping, they would be guaranteed to be able to reject any correct null hypothesis at any Type I error level!) In contrast, the post-experimental odds approach we recommend is not susceptible to biasing via optimal stopping (cf. Berger, 1985 and Berger & Berry, 1988).

The reason that the post-experimental odds do not depend on optional stopping is that the effect of optional stopping on the likelihood of observing some realization of the data is a multiplicative constant. When one considers the odds of one model to another, this same constant is present in the likelihood of each model, and hence it cancels when taking the ratio of the likelihoods. Thus, the Bayesian interpretation of post-experimental odds is unaffected by optional stopping. And since the post-experimental odds have complete frequentist justification, the frequentist who employs them can also ignore optional stopping.

In Section 6 we put forward our recommendations for statistical practice in testing. These recommendations can be boiled down to two: researchers should report the pre-experimental rejection ratio when presenting their experimental design, and researchers should report the post-experimental rejection ratio when presenting their results. How exactly these recommendations should be adopted will vary according to the standard practice in the science. For some sciences there are nothing but *p*-values; for others, there are also minimal power considerations; for some there are sophisticated power considerations; and for some there are full blown specifications of prior odds ratios and prior distributions. Our recommendations accommodate all.

Most of the ideas in this paper have precedents in prior work. What we call the ‘pre-experimental rejection odds’ is nearly identical to Wacholder et al.’s (2004) ‘false-positive reporting probability,’ as further developed by Ioannidis (2005) and the Wellcome Trust Case Control Consortium (2007). The ‘postexperimental rejection odds’ approach is known in the statistics literature as Bayesian hypothesis testing. For psychology research, there have been advocates for Bayesian analysis in general (e.g., Kruschke, 2011) and for Bayesian hypothesis testing in particular (e.g., Masson, 2011; Wagenmakers et al., in press). The Bayes factor bound was introduced by Sellke et al. (2001) and Vovk (1993). We view our contribution primarily as presenting these ideas to the research community in a unified framework and in terms of actionable changes in statistical practice. As noted above, however, as far as we know, our frequentist justification for Bayes factors is a new result and helps to unify the pre- and post-experimental odds approaches.

## 2. The pre-experimental odds approach: incorporating the anticipated effect size and prior odds into interpretation of statistical significance

Rejecting the null hypothesis at the 0.05 significance threshold is typically taken to be sufficient evidence to accept the alternative hypothesis. Such reasoning, however, is erroneous for at least two reasons.

First, what one should conclude from statistical significance depends not only on the probability of statistical significance under the null hypothesis – the significance threshold of 0.05 – but also on the probability of statistical significance under the alternative hypothesis – the power of the statistical test. Second, the prior odds in favor of the alternative hypothesis is relevant for the strength of evidence that should be required. In particular, if the alternative hypothesis would have seemed very unlikely prior to running the experiment, then stronger evidence should be needed to accept it. This section develops a more accurate way to interpret the evidentiary impact of statistical significance that takes into account these two points.

### 2.1. Pre-experimental rejection odds: the correct way to combine type I error and power

Recall that we are interested in testing the null hypothesis, *H*_0_ : *θ* = 0, against the alternative hypothesis, *H*_1_ : *θ* ≠ 0. Both standard frequentist and Bayesian approaches can be expressed through choice of a prior density *π*(*θ*) of *θ* under *H*_1_. To a frequentist, this prior distribution represents a ‘weight function’ for power computation. Often, this weight function is chosen to be a point mass at a particular anticipated effect size, i.e., the power is simply evaluated at a fixed value of *θ*.

Given the test statistic for the planned analysis (for example, the *t*-statistic), the rejection region ℛ is the set of values for the test statistic such that the null hypothesis is said to be ‘rejected’ and the finding is declared to be statistically significant. The ‘significance threshold’ *α* (the Type I error probability) is the probability under *H*_0_ that the test statistic falls in the rejection region. In practice, the rejection region is determined by the choice of the significance threshold, which is fixed, typically at *α* = 0.05. Given the experimental design and planned analysis, the Type I error probability *α* pins down a Type II error probability *β*(*θ*) for each possible value of *θ*: the probability that the test statistic does *not* fall in the rejection region when the parameter equals *θ*. The average power is (1 − *β̄*) ≡ ∫ (1 − *β*(*θ*))*π*(*θ*)*dθ* (which, again, could simply be the power evaluated at a chosen fixed effect size).

We want to know: if we run the experiment, what are the odds of *correct* rejection of the null hypothesis to *incorrect* rejection? We call this quantity the ‘pre-experimental rejection odds’ (sometimes dropping the word ‘rejection’ for brevity). Given the definition of the Type I error probability *α*, the probability of incorrectly rejecting *H*_0_ is *π*_0_*α*, where *π*_0_ is the (prior) probability that *H*_0_ is true. Given the definition of average power 1 − *β̄*, the probability of correctly rejecting *H*_0_ is *π*_1_(1 − *β̄*), where *π*_1_ = 1 − *π*_0_ is the (prior) probability that *H*_1_ is true. The following definition takes the odds that result from these quantities and slightly reorganizes the terms to introduce key components of the odds.

#### Definition

The *pre-experimental odds of correct to incorrect rejection of the null hypothesis* is

(2) $$O_{\mathrm{pre}}=\frac{\pi_{1}}{\pi_{0}}\times \frac{\left(1-\bar{\beta }\right)}{\alpha }$$

(3) $$\equiv O_{P}\times R_{\mathrm{pre}}\;\equiv [\text{prior}\;\text{odds}\;\text{of}\;H_{1}\;\text{to}\;H_{0}]\times [(\text{pre-experimental})\;\text{rejection}\;\text{ratio}\;\text{of}\;H_{1}\;\text{to}\;H_{0}].$$

An alternative definition of the pre-experimental odds provides a Bayesian perspective: *O_pre_* could be defined as the odds of *H*_1_ to *H*_0_ conditional on the finding being statistically significant: $O_{\mathrm{pre}}\equiv \frac{\mathrm{Pr}(H_{1}|R)}{\mathrm{Pr}(H_{0}|R)}$. Bayes’ Rule then implies that $\frac{\mathrm{Pr}(H_{1}|R)}{\mathrm{Pr}(H_{0}|R)}=\frac{\mathrm{Pr}(H_{1})}{\mathrm{Pr}(H_{0})}\frac{\mathrm{Pr}(R|H_{1})}{\mathrm{Pr}(R|H_{0})}=\frac{\pi_{1}}{\pi_{0}}\times \frac{\left(1-\bar{\beta }\right)}{\alpha }$, as above. Of course, this would be the Bayesian answer only if the information available was just that the null hypothesis was rejected (i.e., *p*-value < *α*); as we will see, if the *p*-value is known, the Bayesian answer will differ.

The fact that *O_pre_* arises as the product of the prior odds and rejection ratio is scientifically useful, in that it separates prior opinions about the hypotheses (which can greatly vary) from the *pre-experimental rejection ratio*, *R_pre_*, provided by the experiment; this latter is the odds of rejecting the null hypothesis when *H*_1_ is true to rejecting the null hypothesis when *H*_0_ is true.

**Fig. 1:**
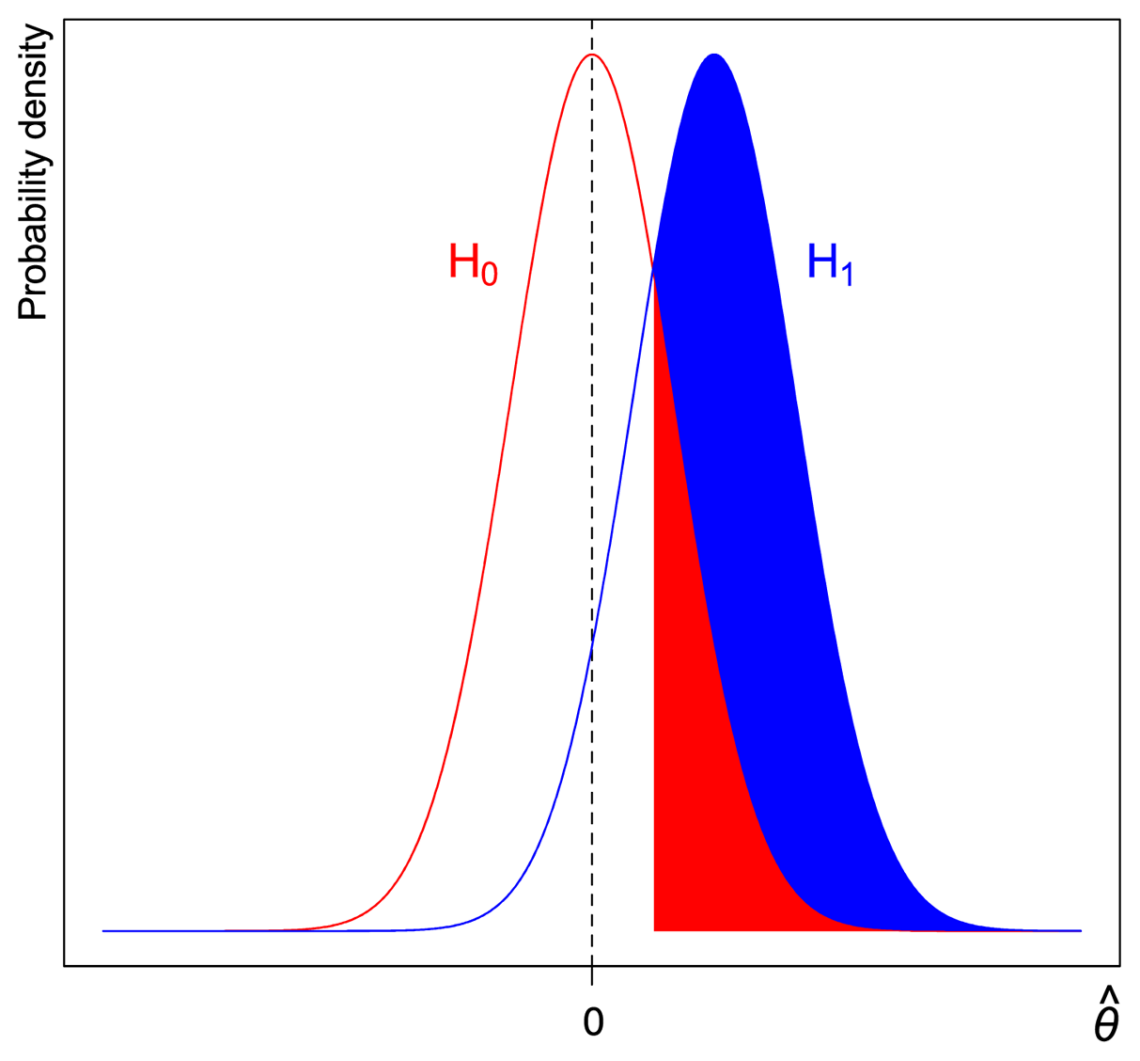
The probability density of the observed effect under the null model (red curve) and a point alternative (blue curve). The area of the red shaded region, which equals 0.05, is the probability of observing a statistically significant result under *H*_0_. The area of the blue region plus the area of the red region is the probability of observing a statistically significant result under *H*_1_, which equals the level of statistical power.

Fig. 1 illustrates how the ratio of power to the significance threshold (the rejection ratio) represents the evidentiary impact of statistical significance. Under the null hypothesis *H*_0_ : *θ* = 0, the red curve shows the probability density function of the estimated effect *θ̂*, assumed to have a normal distribution. The red shaded region on the right tail shows the one-sided 5% significance region. (We focus on the one-sided region merely to simplify the figure.) The area of the red shaded region, which equals 0.05, is the probability of observing a statistically significant result under *H*_0_. The blue curve shows the probability density function of the estimated effect *θ̂* under a point alternative hypothesis *H*_1_ : *θ* = *θ*_1_ > 0. The area of the blue region plus the area of the red region is the probability of observing a statistically significant result under *H*_1_, which equals the level of statistical power. Their ratio, $\frac{\text{red}\;\text{area}+\text{blue}\;\text{area}}{\text{red}\;\text{area}}=\frac{1-\bar{\beta }}{\alpha }$, is the rejection ratio.

The rejection ratio takes into account the crucial role of power in understanding the strength of evidence when rejecting the null hypothesis, and does so in a simple way, reducing the evidence to a single number. Table 1 shows this crucial role. For example, in a low powered study with power equal to only 0.25, *α* = 0.05 results in rejection ratio of only 5 : 1, which hardly inspires confidence in the rejection. Researchers certainly understand that calculating power prior to running an experiment is valuable in order to evaluate whether the experiment is sufficiently likely to ‘work’ to be worth running in the first place. Once an effect is found to be statistically significant, however, there is a common but faulty intuition that statistical power is no longer relevant.^2^

Calculating the rejection ratio requires knowing the power of the statistical test, which in turn requires specifying an anticipated effect size or more generally a prior distribution over effect sizes *π*(*θ*). Of course, choosing an anticipated effect size can be tricky and sometimes controversial; see Gelman and Carlin (2014) for some helpful discussion of how to use external information to guide the choice. We advocate erring on the conservative side (i.e., assuming ‘too small’ an effect size) because many of the relevant biases in human judgment push in the direction of assuming too large an effect size. For example, researchers may be subject to a ‘focusing illusion’ (Schkade & Kahneman, 1998), exaggerating the role of the hypothesized mechanism due to not thinking about other mechanisms that also matter. As another example, since obtaining a smaller sample size is usually less costly than a larger sample size, researchers may wishfully convince themselves that the effect size is large enough to justify the smaller sample.

We also caution researchers against uncritically relying on meta-analyses for determining the anticipated effect size. There are three reasons. First, there may be publication bias in the literature due to the well known ‘file drawer problem’ (Rosenthal, 1979): the experiments that did not find an effect may not be published and thus may be omitted from the meta-analyses, leading to an upward bias in the estimated effect size. Second and relatedly, if experiments that find a significant effect are more likely to be included in the analysis, then the estimated effect size will be biased upward due to the ‘winner’s curse’ (e.g., Garner, 2007): conditional on statistical significance, the effect estimate is biased away from zero (regardless of the true effect size). Third and finally, if any of the studies included in the meta-analysis adopted questionable research practices that inflate the estimated effects (see John et al., 2012) or practices that push estimated effects toward the null (such as using unusually noisy dependent variables), then the meta-analysis estimate will be correspondingly biased.

#### Example 1 (The Effect of Priming Asian Identity on Delay of Gratification)

In an experiment conducted with Asian–American undergraduate students, Benjamin, Choi, and Strickland (2010) tested whether making salient participants’ Asian identity increased their willingness to delay gratification. Participants in the treatment group (*n* = 37) were asked to fill out a questionnaire that asked about their family background. Participants in the control group (*n* = 34) were asked instead to fill out a questionnaire unrelated to family background. In both groups, after filling out the questionnaire, participants made a series of choices between a smaller amount of money to be received sooner (either today or in 1 week) and a larger amount of money to be received later (either in 1 week or in 2 weeks). The research question was how often participants in the treatment group made the patient choice relative to participants in the control group.

What was the pre-experimental rejection ratio *R_pre_* for this experiment?^3^ A conservative anticipated effect size may be *d* = 0.26, where ‘Cohen’s *d*’ is the difference in means across treatment groups in standard-deviation units (a common effect-size measure for meta-analyses in psychology). This value was the average effect size reported in a meta-analysis of explicit semantic priming effects (Lucas, 2000), such as the effect of seeing the word ‘doctor’ on the speed of subsequent judgments about the conceptually related word ‘nurse.’ Given that hypothetical effect size and the actual sample sizes, the power of the experiment was 0.19. Thus *R_pre_* was only $\frac{0.19}{0.05}=3.8$.

What rejection ratio should be considered acceptable? One answer is implicit in the conventions for significance threshold (0.05) and acceptable power (0.80). In that case, the rejection ratio is 16 : 1. While choosing a threshold for an ‘acceptable’ rejection ratio is somewhat arbitrary, to maintain continuity with existing conventions, we will adopt a threshold of 16 : 1 for ordinary circumstances (but we will discuss circumstances when a different threshold is warranted in the next subsection).Planned sample sizes should be sufficient to ensure an adequate rejection ratio. If the rejection ratio of the planned experiment is too small, then the experiment is not worth running because even a statistically significant finding does not provide much information. The directive to plan for a rejection ratio of 16 : 1 will often be equivalent to the usual directive to plan for 80% power.

Unfortunately, current norms in many sciences often lead to *much* less than 80% power. Indeed, low power appears to be a problem in a range of disciplines, including psychology (Cohen, 1962, 1988; Vankov, Bowers, & Munafò, 2014), neuroscience (Button et al., 2013), and experimental economics (Zhang & Ortmann, 2013). To illustrate, we use Richard, Bond, and Stokes-Zoota’s (2003) review of meta-analyses across a wide range of research topics in social psychology. Averaged across research areas, they estimate a ‘typical’ effect size of *r* = 0.21, where *r* is the Pearson product–moment correlation coefficient between the dependent variable and a treatment indicator (another common effect-size measure for meta-analyses in psychology). Given this effect size, Table 2 shows statistical power and the rejection ratio at the 0.05 significance threshold for an experiment conducted with different sample sizes. For simplicity, we assume that each observation is drawn from a normal distribution with unit variance. For the control group, the mean is 0, while for the treatment group, the mean is 0.21. We assume that the treatment and control group each have a sample size of *n*.

In some areas of psychology, typical sample sizes are as small as *n* = 20 participants per condition, and in many fields, typical sample sizes are smaller than 50 per condition. Given an effect size of *r* = 0.21, however, 280 participants per condition are needed for 80% power. Of course, there are substantial differences in typical effect sizes across research areas, and in any particular case, the power calculations should be suited to the appropriate anticipated effect size.

### 2.2. Setting the significance threshold α

By convention, in psychological research and many other sciences, the statistical significance threshold *α* is almost always set equal to 0.05. Thinking about the pre-experimental odds sheds light on why 0.05 is often *not* an appropriate significance threshold, and it provides a framework for determining a more appropriate level for *α*. (While we highly recommend scientists consider tailoring the significance threshold to reflect the prior odds, this subsection can be skipped, without loss of continuity, by those who do not want to consider prior odds.)

Recall that the pre-experimental odds depend not only on the rejection ratio, but also the prior odds: $O_{\mathrm{pre}}=\frac{\pi_{1}}{\pi_{0}}\times \frac{\left(1-\bar{\beta }\right)}{\alpha }$. A statistical test that has rejection ratio of 16 : 1 has pre-experimental odds of 16 : 1 only if, prior to the experiment, *H*_1_ and *H*_0_ were considered equally likely to be true. If *H*_1_ has a much lower prior probability than *H*_0_, say the prior odds are less than 1 : 16, then the pre-experimental odds are less than one even if *p <* 0.05.

In fact, since power can never exceed 100%, when the significance threshold is 0.05, the largest possible rejection ratio is 1 : 0.05 = 20 : 1. Therefore, when *α* = 0.05, if the prior odds are less than 1 : 20, the null hypothesis remains more likely than the alternative hypothesis even when the result is in the rejection region.

#### Example 2 (Evidence for Parapsychological Phenomena)

In a controversial paper, Bem (2011) presented evidence in favor of parapsychological phenomena from 9 experiments with over 1000 participants. There have been many criticisms of this paper. Wagenmakers, Wetzels, Borsboom, and van der Maas’ (2011) ‘Problem 2’ can be understood in terms of the pre-experimental odds framework presented here. While it is of course highly speculative to put a prior probability on the existence of parapsychological phenomena, for illustrative purposes Wagenmakers et al. assume *π*_1_ = 10^−20^ (and *π*_0_ = 1 − *π*_1_). With such a skeptical prior probability, what is the evidentiary impact of statistical significance at the 0.05 threshold? Not much. Since the rejection ratio is bounded above by 20 : 1, the pre-experimental odds can be at most $\frac{10^{-20}}{1-10^{-20}}\frac{20}{1}\approx 2\times 10^{-19}$.

When the prior odds are low, the significance threshold needs to be made more stringent in order for statistical significance to constitute convincing enough evidence against the null hypothesis.

#### Example 3 (Genome-Wide Association Studies)

Early genomic epidemiological studies had a low replication rate because they were conducting hypothesis tests at standard significance thresholds. In 2007, a very influential paper by the Wellcome Trust Case Control Consortium proposed instead a cutoff of *p <* 5×10^−7^. The argument for this was a pre-experimental odds argument. Using the earlier notation, they argued that $O_{P}=\frac{1}{100,000}$, assumed that (1 − *β̄*) = 0.5, and wanted pre-experimental odds of 10 : 1 in order to claim a discovery. Solving for *α* yields *α* = 5×10^−7^. Using this criterion, the paper reported 21 genome/disease associations, virtually all of which have been replicated.

Subsequent work tightened the significance threshold further, and the current convention for ‘genome-wide significance’ is *α* = 5 × 10^−8^. Genome-wide association studies using this threshold have continued to accumulate a growing number of robust findings (Rietveld et al., 2014; Visscher, Brown, McCarthy, & Yang, 2012).

Of course, adopting a more stringent significance threshold than 0.05 will mean that, for a given anticipated effect size, attaining an adequate level of statistical power will require larger sample sizes – perhaps *much* larger sample sizes. Indeed, recent genomewide association studies that focus on complex traits (influenced by many genetic variants of small effect), such as height (Wood et al., 2014), obesity (Locke et al., 2015), schizophrenia (Ripke et al., 2014), and educational attainment (Rietveld et al., 2013), have used sample sizes of over 100,000 individuals.

We suspect that our examples of parapsychological phenomena and genome-wide association studies are extreme within the realm of experimental psychology; most domains will not have prior odds quite so stacked in favor of the null hypothesis. Nonetheless, we also suspect that many domains of experimental psychology should adopt significance thresholds more stringent than 0.05 and should generally feature studies with larger sample sizes than are currently standard.

## 3. The post-experimental rejection odds approach: finding the rejection odds corresponding to the observed data

While pre-experimental rejection odds (and the pre-experimental rejection ratio) are relevant prior to seeing the data, their use after seeing the data has been rightly criticized by many (e.g., Lucke, 2009). After all, the pre-experimental rejection ratio for an *α* = 0.05-level study might be 16 : 1, but should a researcher report 16 : 1 regardless of whether *p* = 0.05 or *p* = 0.000001?

One of the main attractions in reporting *p*-values is that they measure the strength of evidence against the null hypothesis in a way that is data-dependent. But reliance on the *p*-value tempts researchers into erroneous interpretations. For example, *p* = 0.01 does *not* mean that the observed data had a 1% chance of occurring under the null hypothesis; the correct statement is that, under the null hypothesis, there is a 1% chance of a test statistic as extreme or more extreme than what was observed. And when correctly interpreted, the *p*-value has some unappealing properties. For example, it measures the likelihood that the data would be more extreme than they were, rather than being a measure of the data that were actually observed. The *p* = 0.01 also focuses exclusively on the null hypothesis, rather than directly addressing the (usually more interesting) question of how strongly the evidence supports the alternative hypothesis relative to the null hypothesis.

In this section, we present the post-experimental rejection odds. Like their pre-experimental cousin discussed in the last section, the post-experimental odds focus on the strength of evidence for the alternative hypothesis relative to the null hypothesis. However, the post-experimental odds are data-dependent and have a straightforward interpretation as the relative probability of the hypotheses given the observed data.

### 3.1. Post-experimental odds

**Fig. 2:**
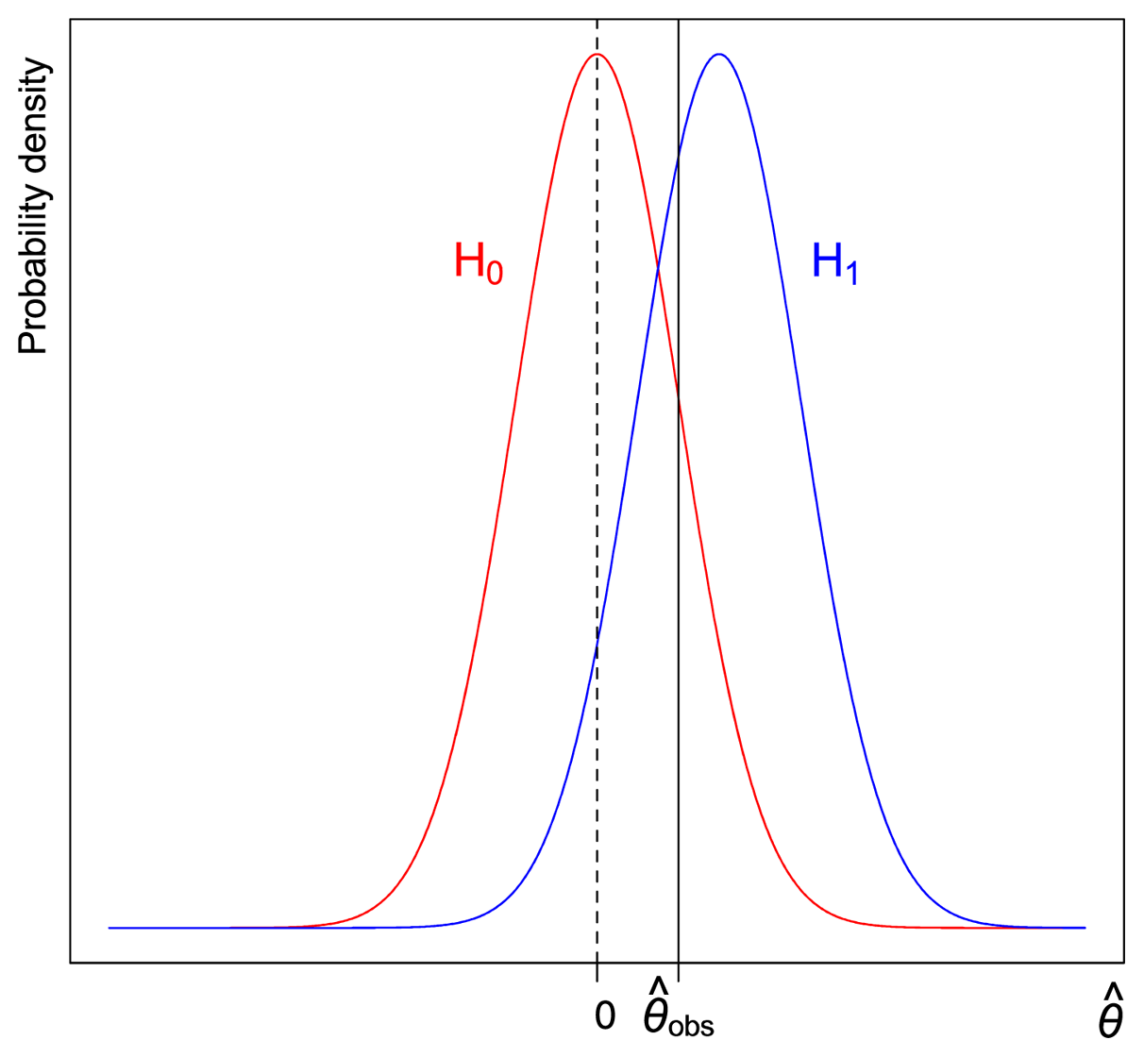
The post-experimental rejection ratio (Bayes factor) is the ratio of the probability density of *θ̂_obs_* under *H*_1_ to the probability density of *θ̂_obs_* under *H*_0_.

The *post-experimental rejection odds* (also called posterior odds) of *H*_1_ to *H*_0_ is the probability density of *H*_1_ given the data divided by the probability density of *H*_0_ given the data. These odds are derived via Bayes Rule analogously to the Bayesian derivation of the pre-experimental rejection odds, except conditioning the observed data ***x*** rather than on the rejection region ℛ. The post-experimental odds are given by

(4) $$O_{\mathrm{post}}(x)=\frac{\pi_{1}}{\pi_{0}}\times \frac{m(x)}{f(x|\theta_{0})}\;\equiv O_{P}\times R_{\mathrm{post}}(x),$$

where *R_post_* (***x***) is the *post-experimental rejection ratio* (more commonly called the Bayes factor or weighted likelihood ratio) of *H*_1_ to *H*_0_, and

(5) $$m(x)=\int_{\left\{\theta \neq \theta_{0}\right\}}f(x|\theta )\pi (\theta )d\theta$$

is the marginal likelihood of the data under the prior *π*(*θ*) for *θ* under the alternative hypothesis *H*_1_. Fig. 2 illustrates, in the same context as in Fig. 1, observed data in the rejection region. The post-experimental rejection ratio is the ratio of the probability density under *H*_1_ to the probability density under *H*_0_. Clearly *R_post_* (***x***) depends on the actual data ***x*** that is observed.

We utilize this standard Bayesian framework to discuss post-experimental odds, but note that we will be presenting fully frequentist and default versions of these odds – i.e., versions that do not require specification of any prior distributions.

#### Example 4 (Effectiveness of An AIDS Vaccine)

Gilbert et al. (2011) reports on a study conducted in Thailand investigating the effectiveness of a proposed vaccine for HIV. The treatment consisted of using two previous vaccines, called Alvac and Aidsvax, in sequence, the second as a ‘booster’ given several weeks after the first. One interesting feature of the treatment was that neither Alvac nor Aidsvax had exhibited any efficacy individually in preventing HIV, so many scientists felt that the prior odds for success were rather low. But clearly some scientists felt that the prior odds for success were reasonable (else the study would not have been done); in any case, to avoid this debate we focus here on just *R_post_* (***x***), rather than on the post-experimental odds.

A total of 16,395 individuals from the general (not high-risk) population were involved, with 74 HIV cases being reported from the 8198 individuals receiving placebos, and 51 HIV cases reported in the 8197 individuals receiving the treatment. The data can be reduced to a *z*-statistic of 2.06, which will be approximately normally distributed with mean *θ*, with the null hypothesis of no treatment effect mapping into *H*_0_ : *θ* = 0. If we consider the alternative hypothesis to be *H*_1_ : *θ* > 0, *z* = 2.06 yields a one-sided *p*-value of 0.02.

To compute the pre-experimental rejection ratio, the test was to be done at the *α* = 0.05 level, so ℛ = (1.645,∞) would have been the rejection region for *z*. The researchers calculated the power of the test to be 1 − *β̄* = 0.45, so *R_pre_* = (1 − *β̄*)/*α* = 9. Thus, pre-experimentally, the rejection ratio was 9 : 1, i.e., a rejection would be nine times more likely to arise under *H*_1_ than under *H*_0_ (assuming prior odds of 1 : 1). It is worth emphasizing again that many misinterpret the *p*-value of 0.02 here as implying 50 : 1 odds in favor of *H*_1_, certainly not supported by the pre-experimental rejection ratio, and even less supported by the actual data, as we will see.

Writing it as a function of the *z*-statistic, the Bayes factor of *H*_1_ to *H*_0_ in this example is

$$R_{\mathrm{post}}(z)=\frac{\int_{0}^{\infty }\frac{1}{\sqrt{2\pi }}e^{-{\left(z-\theta \right)}^{2}/2}\pi (\theta )d\theta }{\frac{1}{\sqrt{2\pi }}e^{-{\left(z-0\right)}^{2}/2}},$$

and depends on the choice of the prior distribution *π*(*θ*) under *H*_1_. Here are three interesting choices of *π*(*θ*) and the resulting post-experimental rejection odds:

- Analysis of power considerations in designing the study suggested a ‘study team’ prior,^4^ utilization of which results in *R_post_* (2.06) = 4.0.
- The nonincreasing prior *most favorable* to *H*_1_ is *π*(*θ*) = Uniform(0, 2.95), and yields *R_post_* (2.06) = 5.63. (It is natural to restrict prior distributions to be nonincreasing away from the null hypothesis, in that there was no scientific reason, based on previous studies, to expect any biological effect whatsoever.)
- For *any* prior, *R_post_* (2.06) ≤ 8.35, the latter achieved by a prior that places a point mass at the maximum likelihood estimator of *θ* (Edwards, Lindman, & Savage, 1963).

Thus the pre-experimental rejection ratio of 9 : 1 does not accurately represent what the data says. Odds of 4 : 1 or 5 : 1 in favor of *H*_1_ are indicated when *z* = 2.06, and 9 : 1 is not possible for *any* choice of the prior distribution of *θ*.

### 3.2. A simple bound on the post-experimental rejection ratio (Bayes factor), requiring only the p-value

In this section, for continuity with the statistical literature we draw on, we revert to using the Bayes factor language. Calculating Bayes factors requires some statistical modeling (as illustrated in the above example), which may be a substantial departure from the norm in some research communities. Indeed, one reason for the ubiquitous reporting of *p*-values is the simplicity therein; for example, one need not worry about power, prior odds, or prior distributions. We have argued strongly that consideration of these additional features is of great importance in hypothesis testing but we do not want the lack of such consideration to justify the continued current practice with *p*-values. It would therefore be useful to have a way of obtaining something like the Bayes factor using only the *p*-value. In addition, having such a method would enable assessing the strength of evidence from historical published studies, from which it is often not possible to reconstruct power or prior information.

Here is the key result relating the Bayes factor to the *p*-value:

#### Result 1

Under quite general conditions, if the *p*-value is *proper* (i.e., *p*(***x***) has a uniform distribution under the null hypothesis) and if *p* ≤ 1/*e* ≈ 0.37, then

(6) $$R_{\mathrm{post}}(x)\leq \frac{1}{-\mathrm{ep}\log p}.$$

Note that this bound depends on the data only through the *p*-value (and note that the logarithm is the natural log). The bound was first developed in Vovk (1993) under the assumption that the distribution of *p*-values under the alternative is in the class of *Beta*(1, *b*) distributions. The result was generalized by Sellke et al. (2001), who showed that it holds under a natural assumption on the hazard rate of the distribution under the alternative. Roughly, the assumption (which is implicitly a condition on *π*(*θ*)) is that, under the alternative distribution, $Pr(p<\frac{1}{2}p_{0}|p<p_{0})$ increases as *p*_0_ → 0, so that the distribution of *p* under the alternative concentrates more and more around 0 as one moves close to zero. The bound was further studied in Sellke (2012), who showed it to be accurate under the assumptions made in a wide variety of common hypothesis-testing scenarios involving two-sided testing. For one-sided precise hypothesis testing (e.g. *H*_0_ : *θ* = 0 versus *H*_1_ : *θ* > 0), Sellke (2012) showed that the bound no longer need strictly hold, but that any deviations from the bound tend to be minor.

Although the result provides merely an upper bound on the Bayes factor, it is nonetheless highly useful: we know that the post-experimental rejection ratio can never be larger than this bound. Table 3 shows the value of the Bayes factor bound for *p*-values ranging from the conventional ‘suggestive significance’ threshold of 0.1 to the ‘genome-wide significance’ thresholds mentioned in Example 3.

An important implication of these calculations is that results that just reach conventional levels of significance do not actually provide very strong evidence against the null hypothesis. A *p*-value of 0.05 could correspond to a post-experimental rejection ratio of at most 2.44 : 1. A *p*-value of 0.01 – often considered ‘highly significant’ – could correspond to a post-experimental rejection ratio of at most 8.13 : 1, which falls well short of our standard of 16 : 1.

Although we do not push it in this paper, one could argue that the significance threshold should be chosen so that any result achieving statistical significance constitutes strong evidence against the null hypothesis – especially since, in practice, researchers are tempted to interpret significant results in this way. In that case, the research community may want to change the standard significance threshold from 0.05 to 0.005, a *p*-value that yields a bound on the odds that is close to 16 : 1 rejection odds. Interestingly, this was also the significance threshold proposed in Johnson (2013); the reasoning therein was quite different, but it is telling that various attempts to interpret the meaning of *p*-values are converging to similar conclusions. (And as discussed above, this threshold should be made more stringent if the probability of *H*_1_ is small.)

If the bound is not large, then rejecting the null hypothesis does *not* strongly suggest that the alternative hypothesis is true – but because it is an upper bound, its interpretation when it *is* large is less clear. The following example illustrates.

#### Example 1 (Continued. The Effect of Priming Asian Identity on Delay of Gratification)

Recall from above that, for Benjamin, Choi, and Strickland’s (2010) test of whether making salient participants’ Asian identity increased their willingness to delay gratification, the pre-experimental rejection ratio was only 3.8 : 1. But given what they found, how strong is the evidence against the null hypothesis?

Benjamin et al. reported that participants in the treatment group made the patient choice 87% of the time, compared with 74% of the time in the control group (*t*(69) = 3.43, *p* = 0.001). The Bayes factor bound is thus $\frac{1}{-e\times 0.001\times \log(0.001)}=52.9$. We can conclude that *R_post_* (***x***) ≤ 52.9, but this, by itself, does not allow for a strong claim of significance because it is an upper bound. Indeed, we argue below that, in low-powered studies such as this one, the Bayes factor bound is likely to be far too high.

In such situations, one could, of course, compute the Bayes factor *R_post_* (***x***) explicitly (and again, this will be shown to have complete frequentist justification). But if this cannot be done, we argue for use of the bound as the post-experimental rejection ratio, for two reasons.

**Fig. 3:**
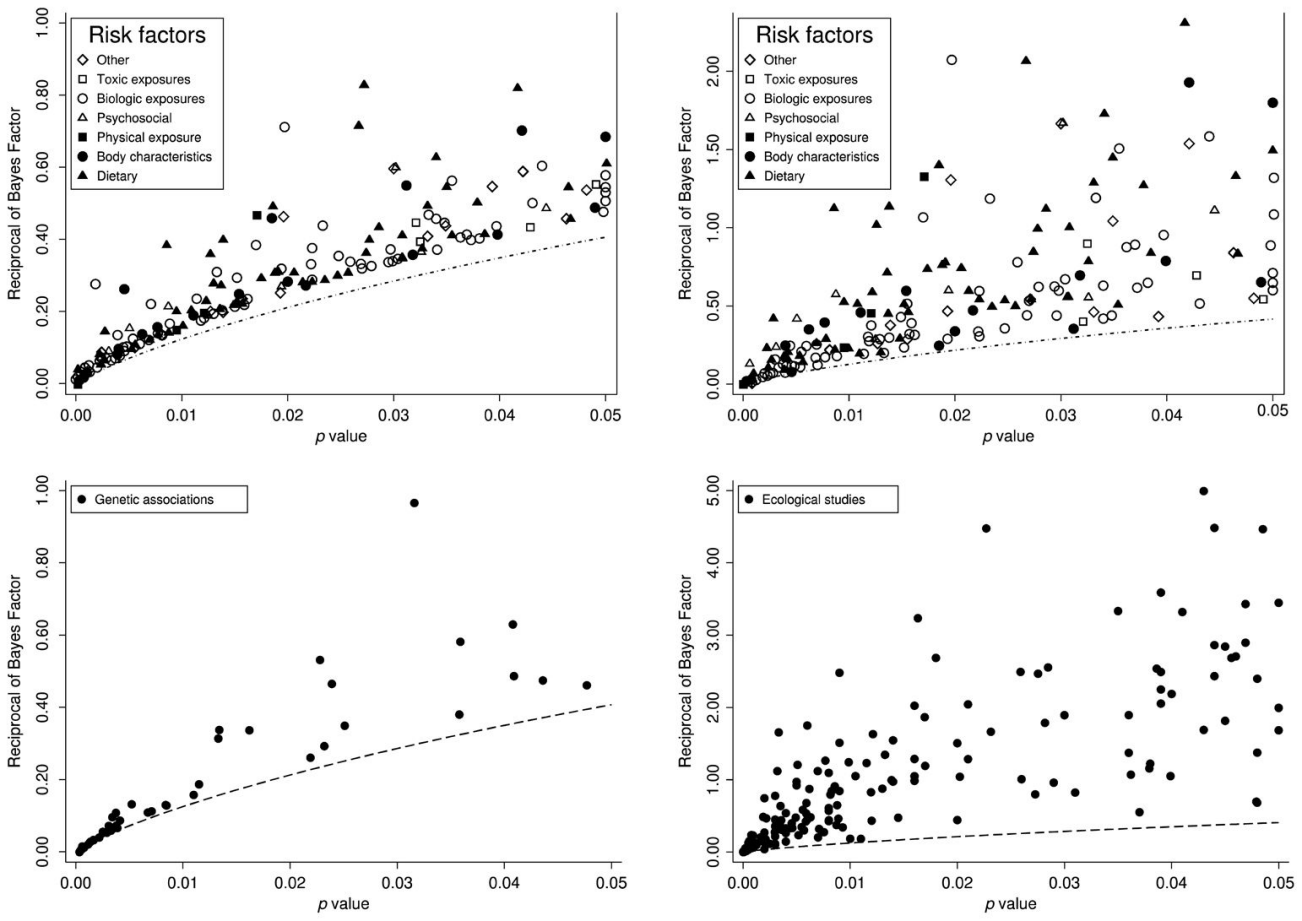
These figures show the relationship between the *p*-value and the reciprocal of the estimated Bayes factor, 1/*R_post_*, for results that were statistically significant (*p <* 0.05) across a range of scientific fields. In each figure, the dashed line shows the reciprocal of the Bayes factor bound (−*ep* log *p*), which is a lower bound for the reciprocal of the Bayes factor. The top two panels graph 1/*R_post_* versus the *p*-value for 272 epidemiological studies; 1/*R_post_* is estimated assuming a relative risk under the alternative of 1.65 in the first panel and 4.48 in the second panel. The lower left panel gives the same for 50 genetic associations; 1/*R_post_* is estimated assuming a relative risk under the alternative of 1.44 (the median observed across the genetic associations). The last panel graphs 1/*R_post_* versus *p*-values for 202 articles published in the journal *Ecology* in 2009; 1/*R_post_* is estimated assuming a standardized effect size under the alternative drawn from Uniform[−6, 6]. This distribution was used in previous work (Sellke et al., 2001), which found that Bayes factor calculations were fairly robust to alternative plausible distributions. The first three panels are Figures 1–3 from Ioannidis (2008). The last panel is an edited version of Figure 4a from Elgersma and Green (2011); of the 308 articles included in the data for that paper, we dropped 6 articles with a *p*-value greater than 0.05, and we dropped 6 additional articles with 1/*R_post_* greater than 5 in order to make the figure more readable.

The first reason is simply that 1/[−*ep* log *p*] is much smaller than 1/*p*, so reporting the former is much better than just reporting *p* and then misinterpreting 1/*p* as being the odds. The second reason is that there is some empirical evidence that indicates that Bayes factors frequently are reasonably close to the bounds. In particular, Fig. 3 displays *p*-values versus the reciprocal of estimated Bayes factors, 1/[*R_post_* ], across studies in a range of scientific fields (these data are from Ioannidis, 2008, and Elgersma & Green, 2011). These Bayes factors have a corresponding *lower bound* equal to [−*ep* log *p*], shown as a hatched curve in all four panels. It can be seen that many of the estimated results lie fairly close to this lower bound.

While these empirical findings are promising, the situation for low powered studies can be considerably worse, as shown in the following example.

#### Example 5 (The Bayes Factor Bound And Power)

An extreme example^5^ of the difference that can arise from low power alternatives is that of observing one observation *X ~ N*(0, *σ*^2^) and testing

$$H_{0}:\sigma^{2}=1\;\;\;\text{versus}\;\;\;H_{1}:\sigma^{2}=1.1.$$

Since the null hypothesis is the usual one, the *p*-value is also just the usual one, e.g., *p* = 0.05 if *x* = 1.96.

Here, for a rejection region of the form |*X*| *≥* 1.96, the power is just 0.0617, so *R_pre_* is only 1.233. The Bayes factor (here, just the likelihood ratio between the hypotheses), for a given *x*, is

$$R_{\mathrm{post}}(x)=(0.953)e^{x^{2}/22}.$$

Table 4 shows the huge discrepancy between the strength of evidence suggested by *p* and the strength of evidence implied by the Bayes factor, but also the large discrepancy between the Bayes factor and 1/[−*e p* log *p*].

The situation improves considerably for higher powered studies. In testing *H*_0_ : *σ*^2^ = 1 versus more ‘separated’ alternatives – in particular the alternative values 4, 9, and 16 – for the same critical region |*X*| *≥* 1.96 and observed *p*-value *p* = 0.05, the pre-experimental rejection ratio is *R_pre_* = 6.54, 10.27, and 12.48, respectively, and the Bayes factors and upper bounds are much closer, as is shown in Table 5.

### 3.3. The surprising frequentist/Bayesian synthesis

The post-experimental odds presented in the previous section was derived as a Bayesian evaluation of the evidence. Surprisingly, this Bayesian answer is also a frequentist answer. To clarify this claim, begin by recalling the frequentist principle.

#### Frequentist principle

*In repeated practical use of a statistical procedure, the long-run average actual accuracy should not be less than (and ideally should equal) the long-run average reported accuracy*.^6^

Here is the key result showing that the post-experimental rejection ratio (Bayes factor) is a valid frequentist report:

#### Result 2

The frequentist expectations of *R_post_* (***x***) and 1/*R_post_* (***x***) over the rejection region are

$$E_{x}[R_{\mathrm{post}}(x)|H_{0},R]=R_{\mathrm{pre}}\;\;\;\text{and}\;E_{x}[1/R_{\mathrm{post}}(x)|H_{1}^{*},R]={\left[R_{\mathrm{pre}}\right]}^{-1}.$$

where $H_{1}^{*}$ refers to the marginal alternative model with density *m*(***x***) (defined in (5)), and the expectations are taken with respect to the sampling distribution of ***x***.

#### Proof

See Appendix

The first identity states that, under *H*_0_, the average of the post-experimental rejection ratios over the rejection region when rejecting (the ‘long-run average reported accuracy’) equals the pre-experimental rejection ratio (the ‘long-run average actual accuracy’). Hence, the frequentist principle is satisfied: if a frequentist reports Bayes factors, then the long-run average will be the pre-experimental rejection ratio.

The second identity is an analogous result that holds under *H*_1_. Whereas the pre- and post-experimental rejection ratios relate to the relative likelihood of *H*_1_ to *H*_0_, the reciprocal of these quantities relate to the relative likelihood of *H*_0_ to *H*_1_. This identity states that the long-run average of the reciprocal of the post-experimental rejection ratio will be the reciprocal of the pre-experimental rejection ratio.

The first identity is completely frequentist, as it involves only the density of the data under the null hypothesis. The second identity, however, is not strictly frequentist because it involves the marginal density of the data (i.e., averaging over all possible nonnull values of *θ* in addition to averaging over the data); the long-run average behavior of *R_post_* (***x***) if *θ* is not null would not be its behavior averaged across values of *θ*, but rather its behavior under the true value of *θ*. For this reason, the discussion hereafter focuses on the first identity.

#### Example 4 (Continued. Effectiveness of An AIDS Vaccine)

**Fig. 4:**
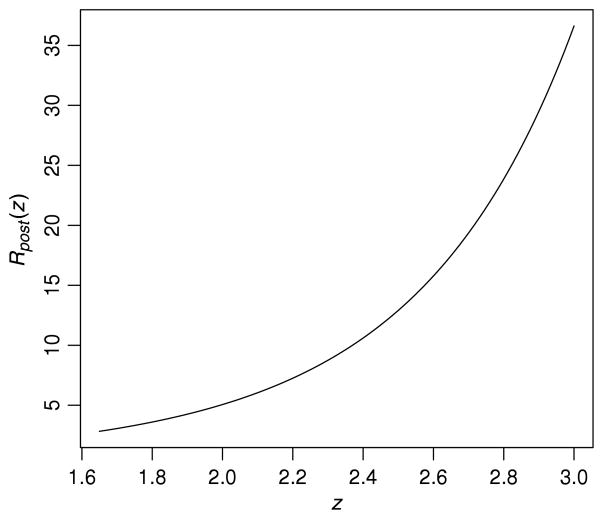
In the vaccine example, *R_post_* (*z*) as a function of *z* over the rejection region.

To illustrate the first identity in Result 2, Fig. 4 presents *R_post_* (*z*) as a function of *z* over the rejection region for Example 4. The value of *R_post_* (*z*) itself ranges from 2 : 1 (for data at the boundary of the rejection region) to ∞. The weighted average of *R_post_* (*z*) as *z* ranges from 1.645 to ∞ (the rejection region) is 9 : 1 (weighted with respect to the density of *z* under the null hypothesis). If one observed *z* = 1.645 (a *p*-value of 0.05) or the actual *z* = 2.06 (a *p*-value of 0.02), the pre-experimental rejection ratio of 9 : 1 would be an overstatement of the actual rejection ratio; if, say, instead, *z* = 3 had been observed, the post-experimental rejection ratio would be 35 : 1, much larger than the pre-experimental rejection ratio.

The possibility that the data could generate post-experimental rejection ratios larger or smaller than the pre-experimental rejection ratio is a logical necessity, as according to Result 2, the latter is an average of the former. Thus logically, the post-experimental rejection ratios must be smaller than the pre-experimental rejection ratio for data near the critical value of the rejection region, with the reverse being true for data far from the critical value.

This frequentist/Bayes equivalence also works for any composite null hypothesis that has a suitable invariance structure,^7^ as long as the alternative hypothesis also shares the invariance structure. As a simple example, it would apply to the very common case of testing the null hypothesis that a normal mean is zero, versus the alternative that it is not zero, when the normal model has an unknown variance. Classical testing in this situation can be viewed as reducing the data to consideration of the *t*-statistic and the noncentral *t*-distribution and testing whether or not the mean of this distribution is zero. As the null hypothesis here is now simple, the above result applies. (More generally, the equivalence follows by reducing the data to what is called the ‘maximal invariant statistic’ for which the null hypothesis becomes simple; see Berger, Boukai, & Wang, 1997, for this reduction in the above example, and Dass & Berger, 2003, for the general invariance theory.)

Result 2 raises a philosophical question: *How can R_post_* (***x***) *be a frequentist procedure if it depends on a prior distribution?* The answer is that *R_post_* (***x***) defines a class of optimal frequentist procedures, indexed by prior distributions. Each prior distribution *π*(*θ*) results in a procedure whose post-experimental rejection ratio equals the pre-experimental rejection ratio in expectation (and, hence, is a valid frequentist procedure); different priors simply induce different power characteristics.

Indeed, *R_post_* (***x***) will tend to be large if the alternative is true and the true value of *θ* is where *π*(*θ*) predicts it to be. Thus, for a frequentist, *π*(*θ*) can simply be viewed as a device to optimally power the procedure in desired locations. Note that these locations need not be where *θ* is believed to be. For instance, a common criterion in classical design is to select a value *θ*_1_ in the alternative that is viewed as being ‘practically different from *θ*_0_’ in magnitude, and then designing the experiment to have significant power at *θ*_1_. For *R_post_* (***x***), one could similarly choose the prior to be centered around *θ*_1_ (or, indeed, to be a point mass at *θ*_1_).

The pre-experimental rejection ratio *R_pre_* is a frequentist rejection-ratio procedure that does not depend on the data; it effectively reports *R_post_* (***x***) to be a constant (e.g., imagine the constant line at 9 in Fig. 4). This procedure, however, is not obtainable from any prior distribution. The reason is that it is the *uniformly worst* procedure, when examined from a conditional frequentist perspective. The intuition behind this claim can be seen from Fig. 4. Any curve that has the right frequentist expectation is a valid frequentist report. Curves that are decreasing in *z* would be nonsensical (reporting lower rejection ratios the more extreme the data), so the candidate curves are the nondecreasing curves. The constant curve (i.e., the pre-experimental rejection ratio) is the worst of this class, as it makes no effort to distinguish between data of different strengths of evidence.

While Result 2 shows that a frequentist is as entitled to report *R_post_* (***x***) as to report *R_pre_*, the logic just outlined shows that *R_post_* (***x***) is clearly a superior frequentist report to *R_pre_*, as it is reflective of the strength of evidence in the actual data, rather than an average of all possible data in the rejection region.

#### Example 3 (Continued. Genome-Wide Association Studies)

Recall the above example of the Wellcome Trust Case Control Consortium, who explicitly calculated the pre-experimental rejection ratio in order to justify their significance threshold. The article also reported the Bayes factors, *R_post_* (***x***), for their 21 discoveries, and the post-experimental odds *O_post_* = *O_P_* × *R_post_* (***x***). These ranged between $\frac{1}{10}$ and 10^68^ for the 21 claimed associations. Thus the post-experimental odds ranged from 1 to 10 against *H*_1_ to overwhelming odds in favor of *H*_1_; reporting these data-dependent odds seems much preferable to always reporting 10 to 1, especially because reporting *R_post_* (***x***) is every bit as frequentist as reporting the pre-experimental rejection ratio.

## 4. Choosing the priors for the post-experimental odds

A potential objection to using the post-experimental rejection odds is that it requires choosing priors: the prior odds $\frac{\pi_{1}}{\pi_{0}}$ and the prior distribution *π*(*θ*) for *θ* under the alternative hypothesis. In the previous section, we addressed philosophical objections to the latter, and we showed that it has fully frequentist justification. In this section, we address the practical question: which priors should a researcher choose?

For the prior odds $\frac{\pi_{1}}{\pi_{0}}$, one option is to report conclusions for a range of plausible prior odds. Another option is to focus the analysis entirely on the Bayes factor, without taking a stand on the prior odds. A Bayesian reader can easily apply his or her own prior odds to draw conclusions.

For choosing the prior distribution *π*(*θ*), here are some options:

1. **Subjective prior**: When a subjective prior is available, such as the ‘study team prior’ in the Example 4, using it is optimal. Again, note that the resulting procedure is still a frequentist procedure with the prior just being used to tell the procedure where high power is desired (as discussed above).
2. **Power considerations**: If the experiment was designed with power considerations in mind, use the implicit prior that was utilized to determine power. This could be a weight function (the same thing as a prior density, but a term preferred by frequentists) if used to compute power, or a specified point (i.e., a prior giving probability one to that point) if that is what was done.
3. **Objective Bayes conventional priors**: Discussion of these can be found in Berger and Pericchi (2001). One popular such prior, that applies to our testing problem, is the *intrinsic prior* defined as follows:
  - Let *π^O^*(*θ*) be a good estimation objective prior (often a constant), with resulting posterior distribution and marginal distribution for data ***x*** given, respectively, by
    $$\pi^{O}(\theta |x)=f(x|\theta )\pi^{O}(\theta )/m^{O}(x),\;m^{O}(x)=\int f(x|\theta )\pi^{O}(\theta )\;d\theta .$$
  - Then the intrinsic prior (which will be proper) is
    $$\pi^{I}(\theta )=\int \pi^{O}(\theta |x^{*})f(x^{*}|\theta_{0})\;dx^{*},$$
    with $x^{*}=(x_{1}^{*},\ldots ,x_{q}^{*})$ being imaginary data of the smallest sample size *q* such that *m^O^*(x*) < ∞.
    *π^I^*(*θ*) is often available in closed form, but even if not, computation of the resulting Bayes factor is often a straightforward numerical exercise.
4. **Empirical Bayes prior**: This is found by maximizing the numerator of *R_post_* (***x***) over some class of possible priors. Common are the class of nonincreasing priors away from *θ*_0_ or even the class of all priors; both were considered in Example 4.
5. *p*-**value bound**: Instead of picking a prior distribution to calculate *R_post_* (***x***), use the generic upper bound on *R_post_* (***x***) that was discussed in Section 3.2.

### Evaluation of these methods

Any of the first three approaches are preferable because they are logically coherent, from both a frequentist and Bayesian perspective. Option 1 is clearly best if either beliefs or power considerations allow for the construction of the prior distribution or power ‘weight function’. Note that there is no issue of ‘subjectivity’ versus ‘objectivity’ here, as this is still a fully frequentist procedure; the prior/power-weight-function is simply being used to ‘place your frequentist bets’ as to where the effect will be. (We apologize to Bayesians who will be offended that we are not separately dealing with prior beliefs and ‘effect sizes’ that should enter through a utility function; we are limited by the scope of our paper.)

Option 2 (the power approach) is the same as Option 1 if a ‘weight function’ approach to power was used. If ‘power at a point’ was done in choosing the design, one is facing boom or bust. If the actual effect size is near the point chosen, the researcher will have maximized post-experimental power; otherwise, one may be very underpowered to detect the effect.

Option 3 (the intrinsic prior approach) is highly attractive if either of the first two approaches cannot be implemented. There is an extensive literature discussing the virtues of this approach (see Berger & Pericchi, 2001, 2015, for discussion and other references).

The last two options above suffer from two problems. First, they are significantly biased (in the wrong way) from both Bayesian and frequentist perspectives. Indeed, if *R̄_post_* (***x***) is the answer obtained from either approach, then

$$R_{\mathrm{post}}(x)<{\bar{R}}_{\mathrm{post}}(x),\;\;\;R_{\mathrm{pre}}<E[{\bar{R}}_{\mathrm{post}}(x)|H_{0},R].$$

Thus, in either case, one is reporting larger rejection ratios in favor of *H*_1_ than is supported by the data.

The (hopefully transient) appeal of using the last two approaches is that they are easy to implement – especially the last. And the answers, even if biased in favor of the alternative hypothesis, are so much better than *p*-values that their use would significantly improve science.

In short, the practical problem of choosing the prior distribution *π*(*θ*) is *not* a compelling argument against the post-experimental odds approach. There are a range of options available, depending on the context and the researchers’ comfort level with statistical modeling. For example, Option 5 is simple and doable in essentially every context, as it avoids the need to specify *π*(*θ*) altogether.

## 5. Post-experimental rejection ratios are immune to optional stopping

A common practice in psychology is to ignore optional stopping (John et al., 2012): if one is close to *p* = 0.05, go get more data to try get below 0.05 (with no adjustment).

### 

#### Example 6 (Optional Stopping)

Suppose one has *p* = 0.08 in a sample of size *n* in testing the null hypothesis that the mean is zero. Suppose the null hypothesis is true. And suppose it is known that the data are drawn from a normal distribution with known variance. If one sequentially takes up to four additional samples of size $\frac{n}{4}$, computing the *p*-value (without adjustment) for the accumulated data at each stage, an easy computation shows that the probability of reaching *p* = 0.05 at one of the four stages (at which point one would stop) is $\frac{2}{3}$. Thus, when using *p*-values to assess significance, optional stopping is cheating. By ignoring optional stopping, one has a large probability of getting to ‘significance.’ (Indeed, if one kept on taking additional samples and computing the *p*-value with no adjustment, one would be guaranteed of reaching *p* = 0.05 eventually, even when *H*_0_ is true.) If one sequentially observes data ***x***_1_, ***x***_2_, … (where each ***x****_i_* could be a single observation or a batch of data), a *stopping rule* ***τ*** is a sequence of indicator functions ***τ*** = (***τ***_1_(***x***_1_), ***τ***_2_(***x***_1_, ***x***_2_), …) which indicate whether or not experimentation is to be stopped depending on the data observed so far. The only technical condition we impose on the stopping rule is that it be *proper*: the probability of stopping eventually must be one.

#### Example 6 (Continued. Optional Stopping)

In the example, ***x***_1_ would be the original data of size *n* and ***x***_2_, … , ***x***_5_ would be the possible additional samples of size *n*/4 each. The stopping rule would be

$$\tau_{1}(x_{1})=\left\{ \begin{array}{ll} 1 & \text{if}\;p(x_{1})<0.05\\ 0 & \text{otherwise}, \end{array}\right.\;\tau_{2}(x_{1},x_{2})=\left\{ \begin{array}{ll} 1 & \text{if}\;p(x_{1},x_{2})<0.05\\ 0 & \text{otherwise}, \end{array}\right.\;\vdots \;\tau_{5}(x_{1},\ldots ,x_{5})=1.$$

An unconditional frequentist *must* incorporate the stopping rule into the analysis for correct evaluation of a procedure – not doing so is really no better than making up data. Thus, for a rejection region ℛ, the frequentist type I error would be *α* = Pr(ℛ | *θ*_0_, ***τ***), the probability being taken with respect to the stopped data density

$$\tau_{k}(x_{1},x_{2},\ldots ,x_{k})f(x_{1},x_{2},\ldots ,x_{k}|\theta_{0}),$$

where *k* denotes the (random) stage at which one stops. Power would be similarly defined, leading to the rejection ratio *R_pre_*, which will depend on the stopping rule.

In contrast, it is well known (cf. Berger, 1985, and Berger & Berry, 1988) that the Bayes factor *does not* depend on the stopping rule. That is, if the stopping rule specifies stopping after observing (***x***_1_, … , ***x****_k_*), the Bayes factor computed using the stopped data density will be identical to that assuming one had a predetermined fixed sample (***x***_1_, … , ***x****_k_*). Intuitively, even though the stopping rule will cause some data to be especially likely to be observed – in particular, data that causes the *p*-value to just cross the significance threshold – the likelihood of observing that data is increased under both the null hypothesis and the alternative hypothesis, leaving the likelihood ratio unaffected. In the formal derivation of the result, the factor ***τ****_k_*(***x***_1_, ***x***_2_, … , ***x****_k_*) appears in both the numerator and denominator of the Bayes factor and therefore cancels out.

There are two consequences of this result:

1. Use of the Bayes factor gives experimenters the freedom to employ optional stopping without penalty. (In fact, Bayes factors can be used in the complete absence of a sampling plan, or in situations where the analyst does not know the sampling plan that was used.)
2. There is no harm if ‘undisclosed optional stopping’ is used, as long as the Bayes factor is used to assess significance. In particular, it is a consequence that an experimenter cannot fool someone through use of undisclosed optional stopping.

#### Example 6 (Continued. Optional Stopping)

Suppose the study reports a *p*-value of 0.05 and no mention is made of the stopping rule. A conventional objective Bayesian analysis will result in a Bayes factor such as *R_post_* = 2. This will certainly not mislead people into thinking the evidence for rejection is strong.

The frequentist/Bayesian duality argument from the previous section still also holds, so that a frequentist can also report the Bayes factor – ignoring the stopping rule – and it is a valid frequentist report. That is, conditional on stopping within the rejection region, the reported ratio of correct to incorrect rejection does not depend on the stopping rule.^8^ This is remarkable and seems like cheating, but it is not. (See Berger, 1985, and Berger & Berry, 1988, for much more extensive discussion concerning this issue.)

To be sure, a frequentist would still need to determine the rejection region ℛ so as to achieve desired Type I and Type II errors and the implied (pre-experimental) rejection ratio. And if one reads an article in which optional stopping was utilized and not reported, one cannot be sure what rejection region was actually used, and so one cannot calculate the pre-experimental rejection ratio. But these are minor points as long as post-experimental rejection ratios are reported; as they do not depend on the stopping rule, the potential to mislead is dramatically reduced.

## 6. Summary: our proposal for statistical hypothesis testing of precise hypotheses

Our proposal can be boiled down to two recommendations: report the pre-experimental rejection ratio when presenting the experimental design, and report the post-experimental rejection ratio when presenting the experimental results. These recommendations can be implemented in a range of ways, from full-fledged Bayesian inference to very minor modifications of current practices. In this section, we flesh out the range of possibilities for each of these recommendations, drawing on the points discussed throughout this paper, in order from smallest to largest deviations from current practice.

### 

#### 1. Report the pre-experimental rejection ratio when presenting the experimental design

This recommendation can be carried out in any research community that is comfortable with power calculations. Reporting the pre-experimental rejection ratio – the ratio of power to Type I error – is a wonderful way to summarize the expected persuasiveness of any significant results that may come out from the experiment.

Of course, calculating power prior to running an experiment has long been part of recommended practice. Our emphasis is on the usefulness of such calculations in ensuring that statistically significant results will constitute convincing evidence. Moreover, beyond conducting power calculations, *reporting* them and the anticipated effect sizes can help ‘keep us honest’ as researchers: knowing that we are accountable to skeptics and critically-minded colleagues encourages us to keep our anticipated effect sizes realistic rather than optimistic.^9^

Moving further away from current practice in many disciplines, we recommend that researchers report their prior odds (for the alternative hypothesis relative to the null hypothesis), or a range of reasonable prior odds.^10^ Research that convincingly verifies surprising predictions of a theory is a major advance and deserves to be more famous and better published. But when the predictions are surprising – that is, when the prior odds in favor of the alternative hypothesis are low – the evidence should have to be more convincing before it is sufficient to overturn our skepticism. That is, when the prior odds are lower, the pre-experimental rejection ratio should be required to be higher in order for the experimental design to be deemed appropriate. At a fixed significance threshold of 0.05, achieving a higher pre-experimental rejection ratio requires running a higher-powered experiment.

To further improve current practice, Type I error of 0.05 should not be a one-size-fits-all significance threshold. When a null hypothesis has a higher prior probability, a more stringent significance threshold should be required for rejecting it. Given the researchers’ prior odds, the appropriate significance threshold can be calculated easily as described in Section 2.2.

#### 2. Report the post-experimental rejection ratio (Bayes factor) when presenting the experimental results

After seeing the data, the pre-experimental rejection ratio should be replaced by its post-experimental counterpart, the Bayes factor: the likelihood of the observed data under the alternative hypothesis relative to the likelihood of the observed data under the null hypothesis. This measure of the strength of the evidence has full frequentist justification and is much more accurate than the pre-experimental measure.

The simplest version of this recommendation is to report the Bayes factor bound: 1/[−*ep* log(*p*)]. Calculating this bound is simple because the only input is the *p*-value obtained from any standard statistical test. Although it only gives an upper bound on what a *p*-value means in terms of the post-experimental ratio of correct to incorrect rejection of the null hypothesis, it is reasonably accurate for well powered experiments. By alerting researchers when seemingly strong evidence is actually not very compelling, reporting of the Bayes factor bound would go far by itself in improving interpretation of experimental results.

Even better is to calculate the actual Bayes factor, although doing so requires some statistical modeling (as illustrated in Example 4) and specification of a ‘prior distribution’ of the effect size under the alternative hypothesis, *π*(*θ*). The frequentist interpretation of *π*(*θ*) is as a ‘weight function’ that specifies where it is desired to have high power for detection of an effect. Hence, if power calculations were used in the experimental design, then the effect size (or distribution of effect sizes) used for the power calculation can be used directly as *π*(*θ*). Other possible ‘objective’ choices for *π*(*θ*) that are well developed in the statistics literature include the intrinsic prior or an empirical Bayes prior.

In research communities in which subjective priors are acceptable, then (as we also recommended in the context of the pre-experimental rejection ratio) researchers should report their prior odds, or a reasonable range, and draw conclusions in light of both the evidence and the prior odds. Indeed, among all of our recommendations, our ‘top pick’ would be to report results in terms of the post-experimental odds of the hypotheses: the product of the prior odds (which may be highly subjective) and the Bayes factor (which is much less subjective). Researchers comfortable with subjective priors could also choose a subjective prior for *π*(*θ*). Of course, to the extent possible, subjective priors – like anticipated effect sizes more generally – should be justified with reference to what is known about the phenomenon under study and about related phenomena, taking into account publication and other biases.

Many of the key parameters relevant for interpreting the evidence – such as anticipated effect sizes, the significance threshold, the pre-experimental rejection ratio, and the prior odds – should be possible to set prior to running the experiment. We therefore further recommend preregistering these parameters. As many have argued, preregistration would help researchers to avoid the hindsight bias (Fischhoff, 1975), not to mention any temptation to tweak the parameters ex post, and hence would make the data analysis more credible.^11^

## Figures and Tables

**Table 1:** (Pre-experimental) rejection ratios for various Type I errors and powers.

Average power *β̄*	0.05	0.25	0.50	0.75	1.0	0.01	0.25	0.50	0.75	1.0
Type I error *α*	0.05	0.05	0.05	0.05	0.05	0.01	0.01	0.01	0.01	0.01
Rejection ratio *R_pre_*	1	5	10	15	20	1	25	50	75	100

**Table 2:** For a fixed effect size of *r* = 0.21, this table shows the statistical power and rejection ratio at the 0.05 significance threshold for an experiment conducted with different sample sizes.

Per-condition *n*	10	20	30	40	50	100	150	200	250	280
Power *βmacr;*	0.12	0.16	0.20	0.24	0.28	0.44	0.57	0.68	0.76	0.80
Type I error *α*	0.05	0.05	0.05	0.05	0.05	0.05	0.05	0.05	0.05	0.05
Rejection ratio *R_pre_*	2.4	3.3	4.1	4.8	5.5	8.7	11.4	13.5	15.2	16.0

**Table 3:** Values of the Bayes factor upper bound for various values of the *p*-value.

*p*	0.1	0.05	0.01	0.005	0.001	0.0001	0.00001	5 × 10^−7^	5 × 10^−8^
$\frac{1}{-\mathrm{ep}\log(p)}$	1.60	2.44	8.13	13.9	52.9	400	3226	2.0 × 10^5^	2.3×10^6^

**Table 4:** For Example 5 (a low powered test), there is a large discrepancy between the strength of evidence suggested by *p* and the strength of evidence implied by the Bayes factor, but also a large discrepancy between the Bayes factor and its upper bound.

*x*	1.65	1.96	2.58	2.81	3.29	3.89	4.42
*p*	0.1	0.05	0.01	0.005	0.001	0.0001	0.00001
*R_post_ (x)*	1.079	1.135	1.290	1.365	1.559	1.897	2.317
1/[−*e p* log *p*]	1.598	2.456	7.988	13.89	53.25	399.4	3195

**Table 5:** In Example 5, the discrepancies between the Bayes factor and the upper bound are considerably reduced for more separated alternatives.

*x*	1.65	1.96	2.58	2.81	3.29	3.89	4.42
*p*	0.1	0.05	0.01	0.005	0.001	0.0001	0.00001
*R_post_ (x), σ* 2 = 4	1.388	2.112	6.067	9.659	28.96	145.7	759.8
*R_post_ (x), σ* 2 = 9	1.118	1.838	6.422	11.14	40.94	277.8	1967
*R_post_ (x), σ* 2 = 16	0.8957	1.513	5.662	10.12	39.94	300.9	2372
1/[−*e p* log *p*]	1.598	2.456	7.988	13.89	53.25	399.4	3195

## HIGHLIGHTS

- Use odds of a correct rejection of the null hypothesis to incorrect rejection.
- Pre-experimentally, these odds are the power divided by the Type I error.
- Post-experimentally, these odds are the Bayes factor.
- The Bayes factor is shown to be a fully frequentist measure of evidence.
- A useful bound on the Bayes factor is given which depends only on the *p*-value.

## Footnotes

1 By *precise hypothesis testing*, we mean that *H*_0_ is a lower dimensional subspace of *H*_1_, as in (1). In particular, the major problem with *p*-values that is highlighted in this paper is muted if the hypotheses are, say, *H*_0_ : *θ* < 0 versus *H*_1_ : *θ* > 0. As an example, suppose *θ* denotes the difference in mean treatment effects for cancer treatments A and B:

- Scenario 1: Treatment A=standard chemotherapy and Treatment B=standard chemotherapy + steroids. This is a scenario of precise hypothesis testing, because steroids could be essentially ineffective against cancer, so that *θ* could quite plausibly be essentially zero.
- Scenario 2: Treatment A = standard chemotherapy and Treatment B = a new radiation therapy. In this case there is no reason to think that *θ* could be zero, and it would be more appropriate to test *H*_0_ : *θ* < 0 versus *H*_1_ : *θ* > 0.

See Berger and Mortera (1999) for discussion of these issues.

2 This line of reasoning – ‘if the evidence is unlikely under the null hypothesis, then the evidence favors the alternative hypothesis’ – may be a version of ‘confirmatory bias’ (see, e.g., Fischhoff & Beyth-Marom, 1983, pp. 247–248). The person making the judgment (in this case, the researcher) is considering only the consistency or inconsistency of the evidence with respect to one of the hypotheses, rather than with respect to both of the possible hypotheses.

3 To be clear, while we conduct this calculation here, and post-experimental calculations in Section 3.2 below, the authors did not report such calculations.

4 This prior distribution was determined for *vaccine efficacy* (VE), which is the percentage of individuals for which the vaccine prevents infection, rather than the simpler parameter *θ* used in the illustration herein. The study team prior density on VE was uniform from −20% (the vaccine could be harmful) to +60%.

5 Our example involves a hypothesis test about the variance of a normal distribution, even though a hypothesis test about the mean would be much more standard in applications. The problem with a hypothesis test about the mean of a normal distribution, such as *H*_0_ : *μ* = 0 versus *H*_1_ : *μ* = 0.25, is that it calls for a one-sided hypothesis test. As noted above, however, the Bayes factor bound need not strictly hold for one-sided tests (Sellke, 2012). While a two-sided test would be common in practical applications, it is unfair to compare such a non-optimal frequentist procedure with the Bayes factor. The basic point of our example – that the Bayes factor bound provides a better approximation to the Bayes factor in higher powered studies – would extend to testing hypotheses about the mean of a normal distribution.

6 Note that our statement of the principle refers here to ‘repeated practical use’. This is in contrast to stylized textbook statements, which tend to focus on the fictional case of drawing new samples and re-running the *same* experiment over and over again. As Neyman himself repeatedly pointed out (see, e.g., Neyman, 1977), the real motivation for the frequentist theory is to provide a *procedure* – for example, rejecting the null hypothesis when *p <* 0.05 – that, if used repeatedly for *different* experiments, would on average have the correct level of accuracy.

7 Here, *invariance* refers to a mathematical theory – concerning transformations of data and parameters in models – that applies when the transformed model has the same structure as the original; see Dass and Berger (2003) for the formal definition within the context of this discussion.

8 This result does *not* apply to the Bayes factor bound in (6), however. That bound assumed that the *p*-value is proper, which does not hold if optional stopping is ignored in its computation.

9 Such reporting would have the additional advantage of facilitating habitual discussion of how the observed effect sizes compared to those that were anticipated and those obtained in related work. Doing so provides information about the plausibility of the observed effect. For example, if the observed effect size is much larger than anticipated, the researcher might be prompted to search for a potential confound that could have generated the large effect.

10 In addition to reporting their own priors, researchers could report the priors of other researchers. For example, it might be useful to report the results of surveying colleagues about what results they expect from the experiment. More ambitiously, prediction markets could be used to aggregate the beliefs of many researchers (Dreber et al., 2015).

11 Many versions of preregistration have been proposed, ranging from researcher-initiated pre-analysis plans that may be deviated from (e.g., Olken, 2015), to journal-enforced preregistration of experimental designs that are peer-reviewed prior to running the experiment (e.g., Chambers, Feredoes, Muthukumaraswamy, & Etchells, 2014). There have also been many thoughtful criticisms of preregistration requirements that may be too rigid or too onerous (e.g., Coffman & Niederle, 2015). Addressing what form preregistration should take and how strictly it should be required or enforced is beyond the scope of this paper.

## Appendix

### Proof of Result 2

We first prove the result for the expected value under the null. Since

$$f(x|H_{0},R)=\frac{f(x|\theta_{0})}{\mathrm{Pr}(R|H_{0})}\;\;\;\text{if}\;x\in R,$$

and 0 otherwise, it follows that

$$E\;[R_{\mathrm{post}}(X)|H_{0},R]=\int \frac{m(x)}{f(x|\theta_{0})}f(x|H_{0},R)dx\;=\frac{1}{\mathrm{Pr}(R|H_{0})}\int_{R}m(x)\;dx\;=\frac{1}{\alpha }\int_{\Theta }\left[\int_{R}f(x|\theta )\;dx\right]\;\pi (\theta )\;d\theta$$

and the result follows trivially by noting that ∫_ℛ_ *f* (***x*** | *θ*) *d****x*** = 1 − *β*(*θ*).

Under the alternative, we consider the testing of *H*_0_ : ***X*** *~ f* (***x*** | *θ*_0_) vs. $H_{1}^{*}:X\sim m(x)$, so now

$$f(x|H_{1}^{*},R)=\frac{f(x|H_{1}^{*})}{\mathrm{Pr}(R|H_{1}^{*})}=\frac{m(x)}{\int_{R}m(x)\;dx}\;\;\;\text{if}\;x\in R,$$

and 0 otherwise; now, as shown above ∫_ℛ_ *m*(***x***) *d****x*** = 1 − *β̄*, so that

$$E\;[1/R_{\mathrm{post}}(x)|H_{1}^{*},R]=\frac{1}{1-\bar{\beta }}\int_{R}\frac{1}{R_{\mathrm{post}}(x)}m(x)\;dx\;=\frac{1}{1-\bar{\beta }}\int_{R}f(x|\theta_{0})\;dx$$

which gives the desired result.
